# Tunable Ni‐Based Alloys as Electrons Donor in Dielectric Isolated Islands to Achieve Broad Frequency Electromagnetic Wave Absorption

**DOI:** 10.1002/advs.202510198

**Published:** 2025-07-16

**Authors:** Pengfei Liu, Da Li, Xiaoguang Zhao, Yanqi Huang, Jingyun Ma, Jiangfang Lian, Shijie Qiu, Zhisen Shen, Zhuan Li, Zhenhui Ma, Song Ma

**Affiliations:** ^1^ The Affiliated Lihuili Hospital of Ningbo University Ningbo 315040 P. R. China; ^2^ Department of Electrical and Electronic Engineering The University of Hong Kong Hong Kong SAR 999077 P. R. China; ^3^ State Key Laboratory of Powder Metallurgy Central South University Changsha 410083 P. R. China; ^4^ Department of Physics Beijing Technology and Business University Beijing 100048 P. R. China; ^5^ Shenyang National Laboratory for Materials Science Institute of Metal Research Chinese Academy of Sciences Shenyang 110016 P. R. China

**Keywords:** alloying treatment, dielectric isolated islands, dielectric loss, electron donating capability, in‐plane interfacial polarization

## Abstract

Nitrogen‐doped carbon (NC) nanosheets with tunable electronic features have been considered as effective dielectric materials to tackle the severe electromagnetic pollution. However, it is still a critical issue to reconcile the contradiction between the skin effect originates from the intrinsic high conductivity of NC and the optimal dielectric loss capability. Herein, we prepared NC nanosheets consisting of numerous microdomain to simultaneously achieve good impedance matching and dielectric dissipation performance. The NC surfaces are separated by defect regions into numerous 50–100 nm dielectric isolated islands, while loading ≈40 nm Ni‐based alloy nanoparticles (NPs) as tunable electron donating sources. The electron cloud density of the loaded NPs can be tuned by altering the alloy species including NiCo, NiFe and NiMn. NiFe and NiMn NPs with stronger electron donating ability can provide more mobile charges in the isolated islands than that of Ni and NiCo NPs, thereby facilitating the enhancement of electron migration and in‐plane interfacial polarization. Consequently, NiFe@NC with better dielectric‐magnetic synergy presented a maximum absorption intensity of −101.6 dB with a broadened absorption bandwidth of 10.7 GHz. This work proposes for the first time to effectively tailor the electron cloud density for absorbers utilizing alloying treatment and offers profound insights into dielectric loss mechanisms.

## Graphical abstract

Tunable electronic sources using Ni‐based alloy in Nitrogen ‐ doped carbon (NC) microdomain are designed to balance the skin effect and the dielectric loss ability. These Ni‐based alloy NPs in dielectric isolated islands can serve as electronic sources to provide abundant mobile electrons to NC nanolayers, thus improving dielectric loss capability in the 2–18 GHz range.

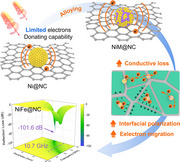

## 1 Introduction

The widespread applications of various intelligent electronic and wireless devices in 5G communication field have brought serious electromagnetic pollution, which puts forward high requirements for electromagnetic wave (EMW) modulation systems to realize strong wideband absorption performance.^[^ ^1^, ^2^, ^3^ ^]^ Generally, the EMW attenuation mechanisms are composed mainly of magnetic loss and dielectric loss, while an effective EMW absorption system should equip the magnetic and dielectric loss ability synchronously to optimize the impendence matching.^[^ ^4^, ^5^, ^6^ ^]^ For the magnetic loss ability, it can be tuned by controlling the magnetic behaviors of magnetic components such as Fe‐based, Co‐based and Ni‐based oxides or metals. However, it is very complex for dielectric loss since this process involves conductivity loss, dipole polarization, interfacial polarization, and so on.^[^ ^7^, ^8^, ^9^ ^]^ Therefore, how to precisely control dielectric loss is still a key challenge.

Among the dielectric materials studied so far, nitrogen‐doped carbon (NC) nanosheets are very attractive due to their excellent electrical features.^[^ ^10^, ^11^, ^12^ ^]^ Furthermore, by compositing magnetic nanoparticles, NC nanosheets can achieve effective magnetic‐electric coupling to optimize electromagnetic wave absorption (EMA) performance.^[^ ^13^ ^]^ However, the excellent conductivity of NC nanosheets leads to significant skin effect, which causes severe reflection of the incident microwave and results in impedance mismatching. One effective strategy to overcome the skin effect is to decrease sample fill ratio to destroy the conductive net, but this would definitely reduce the dielectric loss ability.^[^ ^14^ ^]^ Another available method is to modify functional groups and defects, dope high‐entropy anions or introduce amorphous carbon species on the NC nanosheets in order to reduce the electrical conductivity and inhibit the possible skin effect, which also leads to a poor dielectric loss due to the limited number of mobile electrons.^[^ ^15^, ^16^, ^17^, ^18^ ^]^ Therefore, how to balance the skin effect and the dielectric loss ability to optimize the microwave loss performance has been a key problem in EMA field.

At this regard, we designed tunable electronic sources using Ni‐based alloy in the NC microdomain to well balance the skin effect and the dielectric loss ability. Herein, the 2D NC nanosheets of 5–10 µm were separated into 50–100 nm dielectric isolated islands by defective regions, and ≈40 nm Ni‐based alloyed nanoparticles are randomly distributed in the formed isolated islands and defect regions. Since the defective regions of the as‐prepared NC nanosheets effectively disrupt the conductive network, the possible skin effect would be well impeded. In addition, these Ni‐based alloy NPs in dielectric isolated islands can serve as electronic sources to provide abundant mobile electrons to NC nanolayers, thus improving dielectric loss capability in the 2–18 GHz range.

Moreover, there are a number of in‐plane interfaces between dielectric isolated islands and defect regions, which further promote dielectric responses by interfacial polarizations. In particular, the electrons donating capacity can be tuned by altering the alloy species, such as NiCo, NiFe, and NiMn, to control the number of mobile electrons in dielectric isolated islands and manipulate the induced built‐in electric field. As a result, NiFe and NiMn alloyed NPs with stronger electrons donating capacity exhibit higher dielectric loss features compared to Ni and NiCo NPs with lower mobile electron densities. And thus, NiFe@NC, which combines optimal dielectric characteristics with magnetic loss ability presented excellent EMW absorption performance, with a maximum absorption intensity of −101.6 dB and a broadened absorption bandwidth of 10.7 GHz. This work offers profound insights for efficient electron local movement and contributes to enriching the in‐depth analysis for microwave attenuation mechanisms.

## 2 Results and Discussion

**Figure 1:**
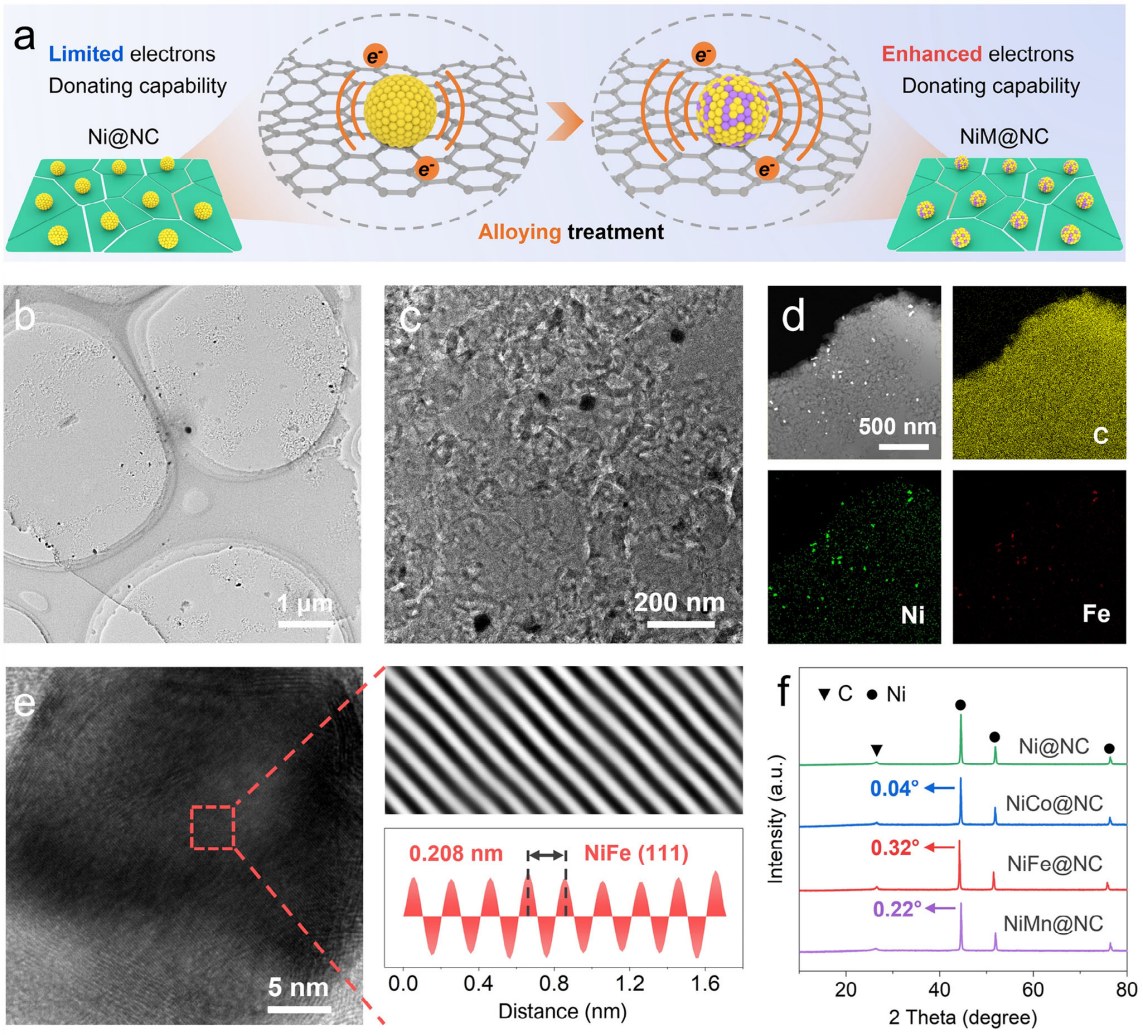
a) Schematic illustration of electron cloud density modulation based on alloying treatment. b,c) TEM images and d) elemental distribution mapping of NiFe@NC. e) HRTEM image of the loaded NiFe particles and the corresponding intensity curve from inverse FFT transformation in the selected zone. f) XRD patterns of the NiM@NC systems.

The schematic diagram of the dielectric isolated islands concept was illustrated in **Figure** **1**a. In this design, the continuous NC substrate was partitioned into isolated nano‐islands by numerous defects, which could effectively disrupt the conductive network to impede skin effect. Correspondingly, the long‐range migration path of electrons would also be interrupted, leading to a significant reduction of dielectric loss ability. To address this issue, the magnetic NPs were loaded on NC nanosheets, serving as electron sources to offer mobile electrons to the isolated regions on the NC matrix, which led to local charge redistribution and promoted polarization responses. Through the introduction of alloying elements with different electronegativity, the electron cloud density of alloyed NPs could be manipulated, thus contributing to the effective control of dielectric properties.

Based on the aforementioned design concept, the 2D nitrogen‐doped carbon nanosheets loading Ni or NiM (M═Co, Fe, and Mn) species (including single‐atom sites and NPs) were prepared by pyrolysis of polydopamine chelated with metal ions, which were denoted as Ni@NC and NiM@NC, respectively. The morphology and microstructure of the prepared composites were revealed by transmission electron microscope (TEM) measurements, which are shown in Figure 1b,c and Figure S1 (Supporting Information). It can be found that the Ni@NC and NiM@NC composites exhibited similar lamellar morphologies. Meanwhile, it can be observed from Figure 1b that the carbon layer surface is quite rough and covered with pitted and undulating defects, which is more evident in Figure 1c. During the carbonization process, the decomposition of nitrate in the metal‐PDA mixture resulted in the generation of large amounts of gases such as NO_2_, NO, and N_2_. The gases destroyed the integrity of the carbon matrix, leading to the formation of numerous defects. Especially, these defective regions separated the carbon layer surface into numerous isolated nano‐islands, thereby breaking the continuity of the interfacial carbon lattice. Consequently, the intrinsic conductive net of carbon nanosheets was disrupted, which contributed to the effective suppression of skin effect induced by high conductivity.

In addition, well‐dispersed particles with an average size of 34.2 nm can be observed on the surface of Ni@NC (Figure S1a, Supporting Information). From Figure 1c and Figure S1b,c (Supporting Information), it can be found that the loaded particles in NiM@NC exhibited comparable size distributions to that of Ni NPs (38.6 nm for NiCo@NC, 36.2 nm for NiFe@NC, and 37.3 nm for NiMn@NC), demonstrating that the alloying did not significantly alter the size of the host particles. Based on the elemental mapping displayed in Figure 1d, the loaded particles can be determined to be Ni‐based alloy NPs. The HRTEM image of the loaded Ni NPs was presented in Figure S2 (Supporting Information). From the intensity profile based on the inversed FFT transformation for selected region, the lattice spacing of particles was determined to be 0.205 nm, which corresponded to the (111) crystal plane of Ni. As shown in Figure 1e, the lattice spacing of NiFe NPs was measured as 0.208 nm, which was slightly expanded than that of Ni NPs.^[^ ^19^, ^20^ ^]^ This result further confirmed the formation of Ni‐based alloy NPs and the success of the alloying treatment.

The X‐ray diffraction (XRD) patterns of the obtained Ni@NC and NiM@NC composites were shown in Figure 1f. The distinct and sharp diffraction peaks in Ni@NC at 44.5°, 51.9° and 76.4° can be attributed to the (111), (200) and (220) crystal planes of metallic nickel, suggesting the well crystallized state of the Ni NPs loaded on the carbon layers. It should be pointed out that the XRD peaks of maximum intensities for NiCo@NC, NiFe@NC and NiMn@NC exhibited a certain degree of negative shifts, indicating the formation of Ni‐based solid solution alloys. As the atomic radius of Co, Fe and Mn are larger than that of Ni atoms, the doping into the Ni matrix leads to an increase in the crystal plane spacing, which results in the smaller diffraction angles and the leftward shifts of the diffraction peaks.^[^ ^21^, ^22^, ^23^ ^]^

**Figure 2:**
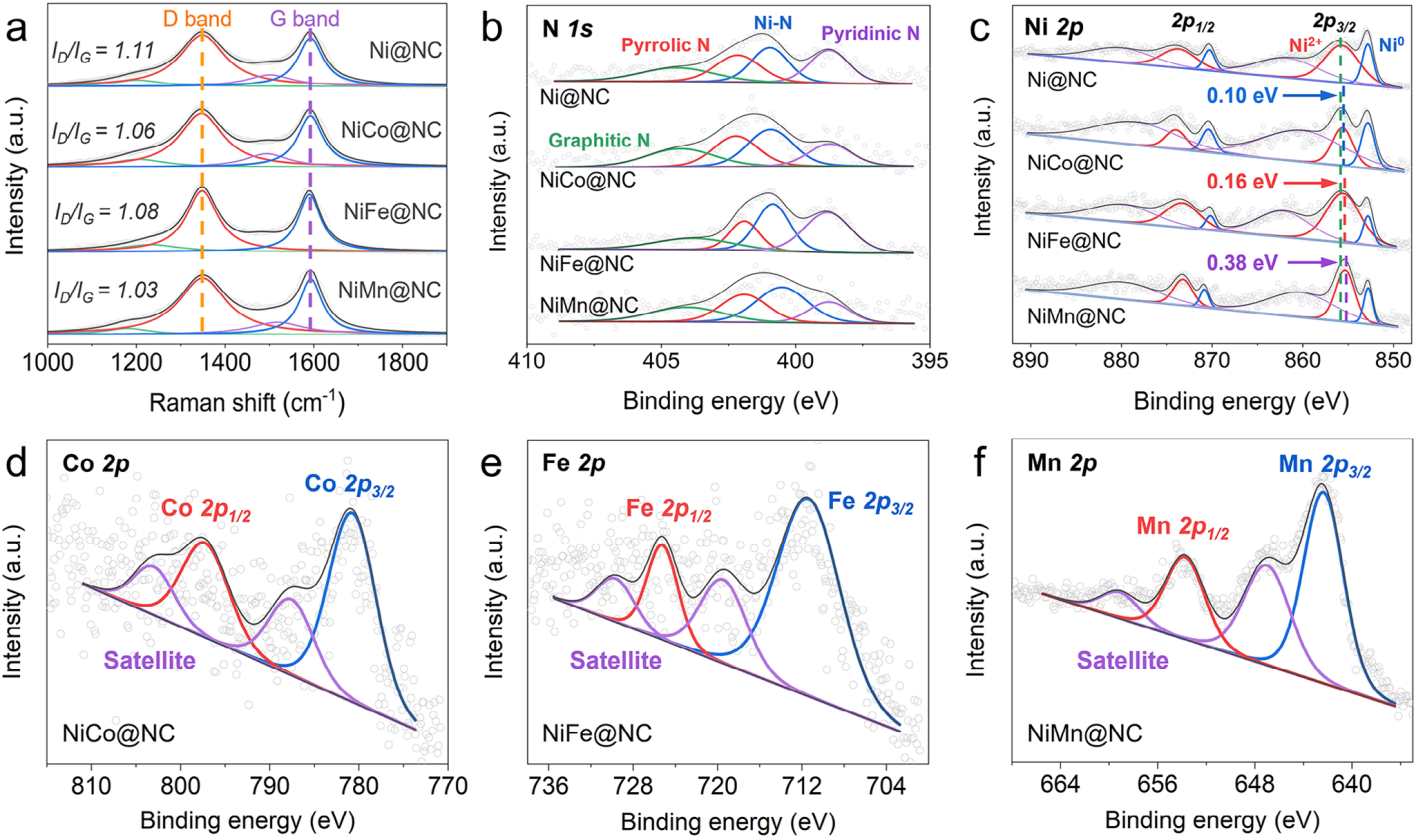
a) Raman spectra, high‐resolution b) N 1s, c) Ni 2p, d) Co 2p, e) Fe 2p, and f) Mn 2p XPS spectra for Ni@NC and NiM@NC composites.

Raman spectra for the as‐prepared Ni@NC and NiM@NC absorbers were recorded in **Figure** **2**a to evaluate the graphitization degree of the NC matrix. The *I_D_/I_G_* values for Ni@NC and NiM@NC composites were all greater than 1.0, indicating the presence of a large number of defects in NC nanosheets, which was consistent with the observation in TEM results. The surface composition and the corresponding chemical states were revealed by X‐ray photoelectron spectroscopy (XPS) measurements. Furthermore, the high‐resolution N 1s spectra in Figure 2b can be deconvoluted into pyridinic N, Ni‐N, pyrrolic N, and graphitic N located at ≈398.8, 400.9, 402.2, and 404.5 eV, respectively.^[^ ^24^, ^25^ ^]^ The emerging Ni‐N peaks manifest that the Ni species in NiM@NC systems were anchored on the carbon substrate via the coordinated N atoms. Besides, Ni‐N signals could also be provided by the Ni single atoms produced on the NC nanosheets. The *d‐p* orbital hybridization between Ni single atoms and NC matrix broke the uniform distribution of local electrons and led to their rearrangements around the N sites, which enhanced the dipole polarization responses and contributed to the strong EMW absorption performance at high frequencies.^[^ ^26^, ^27^, ^28^ ^]^ As illustrated in Figure 2c, the deconvoluted Ni 2p spectra of Ni@NC can be mainly recognized as Ni^0^ (≈852.9 and 870.3 eV), Ni^2+^ (≈855.7 and 873.9 eV), and the broad satellite peaks. The existence of Ni^2+^ can be attributed to the slight oxidation of Ni particles. It is worth noting that the Ni^2+^ peaks of NiM@NC presented negative shifts in varying degrees (0.10 eV for NiCo@NC, 0.16 eV for NiFe@NC, and 0.38 eV for NiMn@NC), suggesting the existence of directional electron migration between NiO and Ni‐based alloy NPs. The shifts toward lower binding energies reveal that Co, Fe, and Mn atoms tend to donate electrons in the alloy NPs, which is consistent with the magnitude of the corresponding atomic electronegativities.^[^ ^29^, ^30^, ^31^ ^]^ Furthermore, as shown in Figure 2d–f, the characteristic peaks of Co, Fe and Mn elements can be observed and deconvoluted into Co 2p_3/2_ (780.9 eV) and Co 2p_1/2_ (797.5 eV),^[^ ^32^ ^]^ Fe 2p_3/2_ (711.5 eV) and Fe 2p_1/2_ (725.2 eV),^[^ ^33^ ^]^ and Mn 2p_3/2_ (642.5 eV) and Mn 2p_1/2_ (653.8 eV),^[^ ^34^ ^]^ respectively. These results confirmed the formation of Ni‐based alloys in NiM@NC absorber systems.

**Figure 3:**
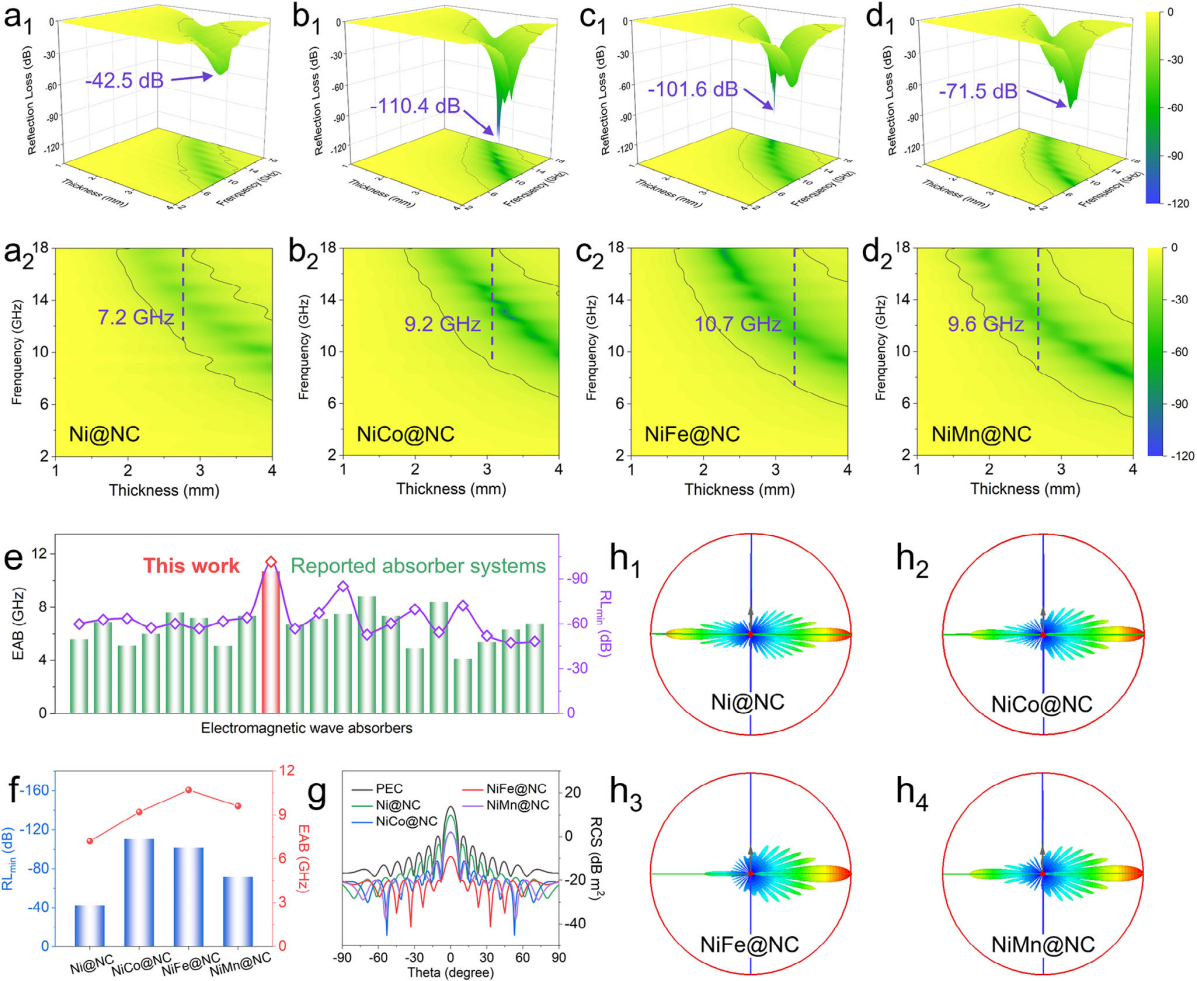
a–d) The 3D plots and 2D contour mapping of reflection loss values for Ni@NC, NiCo@NC, NiFe@NC, and NiMn@NC, respectively. e) Comparison of the EMA performance between NiFe@NC and the reported Ni‐based and electron migration absorber systems. f) Summary of the EMA performance of Ni@NC and NiM@NC absorbers. g) Simulated RCS curves and h) 3D far‐field responses of the as‐prepared NiM@NC absorbers.

The EMA performance of Ni@NC and NiM@NC were assessed on the basis of the coaxial measurement. As shown in **Figure** **3**a, the Ni@NC presented a minimum reflection loss (RL_min_) value of −42.5 dB with an effective absorption bandwidth (EAB) of 7.2 GHz (10.8–18.0 GHz) at 2.8 mm. The alloying process further enhances the EMW absorption performances, as shown in Figure 3b–d. The maximum absorption intensity increased to ‐110.4 dB after the introduction of the Co element, and the EAB also expanded to 9.2 GHz (8.8–18.0 GHz) at a matching thickness of 3.1 mm (Figure 3b). After the incorporation of Fe element, the RL_min_ value of −101.6 dB could be achieved, while the EAB at 3.2 mm further expanded to C band and increased to 10.7 GHz (7.3–18.0 GHz) (Figure 3c). For NiMn@NC, the RL_min_ value was −71.5 dB and the EAB reached 9.6 GHz (8.4–18.0 GHz) at 2.7 mm (Figure 3d). These EMW absorption performances were summarized in Figure 3f, where NiFe@NC exhibited maximal absorption intensity over −100 dB and the largest EAB among all samples. We compared the absorption performance of NiFe@NC with the reported Ni‐based and dielectric loss dominated EMA materials, which exhibited excellent EMW absorption properties in both RL_min_ and EAB values (Figure 3e; Table S2, Supporting Information).

In order to fully evaluate the electromagnetic dissipation performance for the utilization in real‐world scenarios, the far‐field radar cross section (RCS) behavior of Ni@NC and NiM@NC composites were conducted based on CST Studio Suite.^[^ ^16^, ^35^ ^]^ Compared to the perfect electric conductor (PEC) plate, the 3D RCS reflection signals of the as‐prepared absorbers (especially the NiFe@NC) were significantly weakened (Figure 3 h; Figure S6). As shown in Figure 3g, the 2D RCS scattering signals further confirmed the outstanding electromagnetic energy dissipation capacities of Ni@NC and NiM@NC systems. Notably, the RCS values for NiFe@NC were below −10 dB m^2^ when the scanning angles exceeded ± 2° and below −20 dB m^2^ for the scanning angles above ± 10°. Moreover, it can be found in Figure S7 (Supporting Information) that the maximum RCS reduction values of NiM@NC (30.9 dB m^2^ for NiCo@NC, 31.7 dB m^2^ for NiFe@NC, and 24.5 dB m^2^ for NiMn@NC) were much higher than that of Ni@NC (13.2 dB m^2^). Above data indicate that the alloying treatment can effectively enhance the electromagnetic dissipation performance of NiM@NC systems.

**Figure 4:**
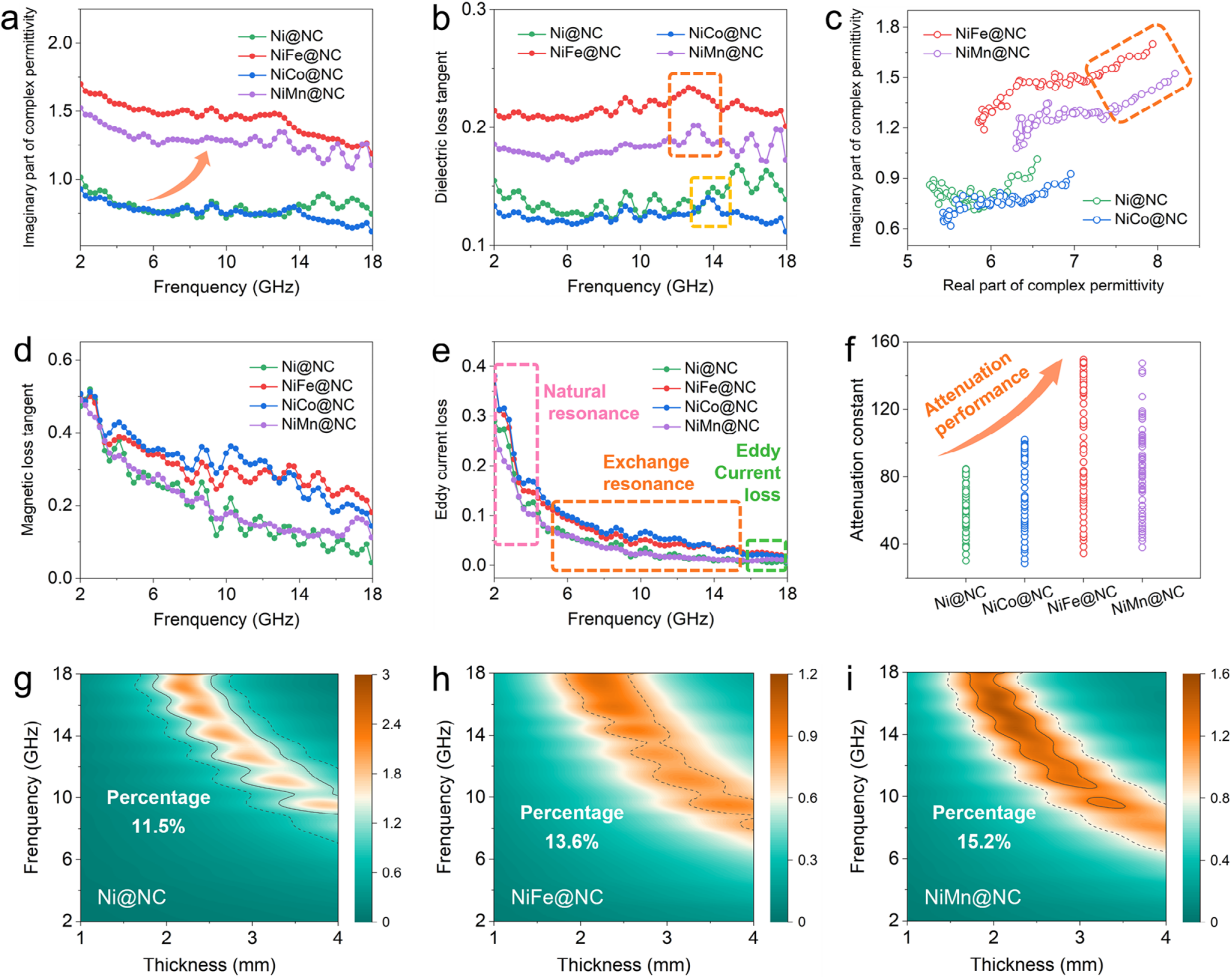
a) Imaginary part of the complex permittivity, b) dielectric loss tangent, c) Cole–Cole curves, d) magnetic loss tangent, e) eddy current loss curves, and f) attenuation constants for the as‐prepared Ni@NC and NiM@NC composites. g–i) 2D impedance matching plots of Ni@NC, NiFe@NC and NiMn@NC, respectively.

To reveal the EMW loss mechanisms in this system, the electromagnetic parameters involving the complex permittivity (*ε_r_* = *ε′* − *jε′′*) and complex permeability (*µ_r_* = *µ′* − *jµ′′*) were shown in **Figure** **4** and Figures S8 and S9 (Supporting Information). As illustrated in Figure S8 (Supporting Information), the real part of the complex permittivity for NiM@NC presented an upward trend compared to that of Ni@NC. Meanwhile, the incorporation of Fe and Mn atoms with lower atomic electronegativity than that of Ni and Co atoms, can contribute to larger ε*′′* values (Figure 4a). As a result, the dielectric loss tangents of NiFe@NC and NiMn@NC were elevated (Figure 4b). The enhanced dielectric loss is mainly attributed to the increased number of mobile electrons since these alloyed samples can provide higher charge density than pure Ni samples. It is worth noting that there is a significant increase in the resonance peak amplitude at ≈13 GHz for NiFe@NC and NiMn@NC compared to that of Ni@NC and NiCo@NC at ≈14 GHz, which may be attributed to the enhanced in‐plane interfacial polarization. NiFe and NiMn NPs with higher electron cloud densities contributed to the charge mobility enhancement, which led to more significant space charge rearrangements at the dielectric microdomain boundaries, inducing a more intense polarization response.

Generally, the Cole–Cole curves can be used to investigate the polarization response of EMA systems. From Figure 4c, it can be seen that the Cole–Cole plots for NiFe@NC and NiMn@NC exhibited semicircles with greater curvature, demonstrating the more adequate Debye relaxation processes.^[^ ^36^ ^]^ In contrast, owing to the relatively close atomic electronegativity with Ni, the Cole–Cole curve of NiCo@NC suggested a comparable dielectric polarization performance to that of Ni@NC. The long and near‐linear tails can be observed from the Cole–Cole curves of NiFe@NC and NiMn@NC, which originated from the conductive loss mainly caused by free charge movement of graphitized carbon matrix under alternating electric‐filed.^[^ ^37^ ^]^ The directional charge transfer along Ni‐N‐C path induced the construction of built‐in electric fields, leading to the localized accumulation of electrons. As a result, the external electric field component drove the long‐range transport and redistribution of carriers from the enriched regions along the carbon lattice, leading to the formation of polarization centers and thus promoting the dielectric losses.^[^ ^13^, ^38^ ^]^

The magnetic loss tangents calculated from *µ′′/ µ′* values were performed to evaluate the magnetic responses of Ni@NC and NiM@NC composites (Figure 4d). NiCo@NC and NiFe@NC exhibited higher *tan δ_µ_* values than that of Ni@NC, while NiMn@NC presented relatively lower *tan δ_µ_* and less resonance peaks, which exhibited a consistent change trend with their saturation magnetization values (*M*_s_), as shown in Figure S5 (Supporting Information). Due to the higher inherent *M*_s_ features of Co and Fe than Ni atoms and the antiferromagnetic feature of Mn atoms, the NiFe@NC and NiCo@NC own more magnetic moments than Ni@NC and NiMn@NC, which contributed to the strong magnetic loss. The C_0_ values (C_0_ = *µ*′′(*µ*′)^−2^ *f* ^−1^) were utilized to explore the detailed magnetic loss mechanisms. As shown in Figure 4e, the multiple resonance peaks located at 2–4 and 5–16 GHz in C_0_ curves for Ni@NC, NiCo@NC, and NiMn@NC can be assigned to natural resonance and exchange resonance, respectively.^[^ ^39^ ^]^ Within 16–18 GHz, the C_0_ values were close to a constant, indicating the presence of eddy current loss. Differently, the C_0_ values of NiMn@NC presented relatively flat fluctuations in 2–12 GHz and were close to a constant from 12–18 GHz. This performance demonstrated that the natural and exchange resonance of NiMn@NC were quite weak, while the eddy current loss dominated within the whole *Ku* band. Considering the enhanced absorption intensity and enlarged effective bandwidth for NiMn@NC, it is reasonable to conclude that the reinforced spatial charge density played a prominent role in boosting the EMW energy conversion as compared to the improvement of magnetic properties for absorber systems. This insight was further supported by the attenuation constants shown in Figure 4f, where the attenuation constant of NiMn@NC in the range of 38.1–147.3 was higher than that of Ni@NC from 30.1 to 84.7 and NiCo@NC within 28.5–101.9. Definitely, the dielectric‐magnetic synergy effect contributes to the further improvement of EMW attenuation performance. As a result, NiFe@NC presented the highest attenuation constant value from 34.7 to 149.5.

The 2D impedance matching plots based on |*Z_in_/Z_0_* | values for Ni@NC and NiM@NC were displayed in Figure 4g–i and Figure S10 (Supporting Information), where the *Z_in_* and *Z_0_* represent the input impedance (the impedance of the absorber) and free space impedance, respectively. When the calculated |*Z_in_/Z_0_* | values are located in the range of 0.8–1.2, it can be considered that the interfacial impedance of absorbers is close to that of the free space, which favors the entry of the incident microwave into the EMA materials to be fully consumed.^[^ ^40^, ^41^ ^]^ The percentage of the matching area in |*Z_in_/Z_0_* | contour mapping for Ni@NC was 11.5%, while that for NiCo@NC, NiFe@NC and NiMn@NC raised up to 13.2%, 13.6% and 15.2%, respectively. Regardless of the increasing or decreasing saturated magnetization degree, the matched percentage for NiCo@NC, NiFe@NC, and NiMn@NC increased in sequence as the electronegativity difference with Ni atoms enlarged, indicating that the optimized electron structure contributed to the adjustment for the interfacial impedance matching.

**Figure 5:**
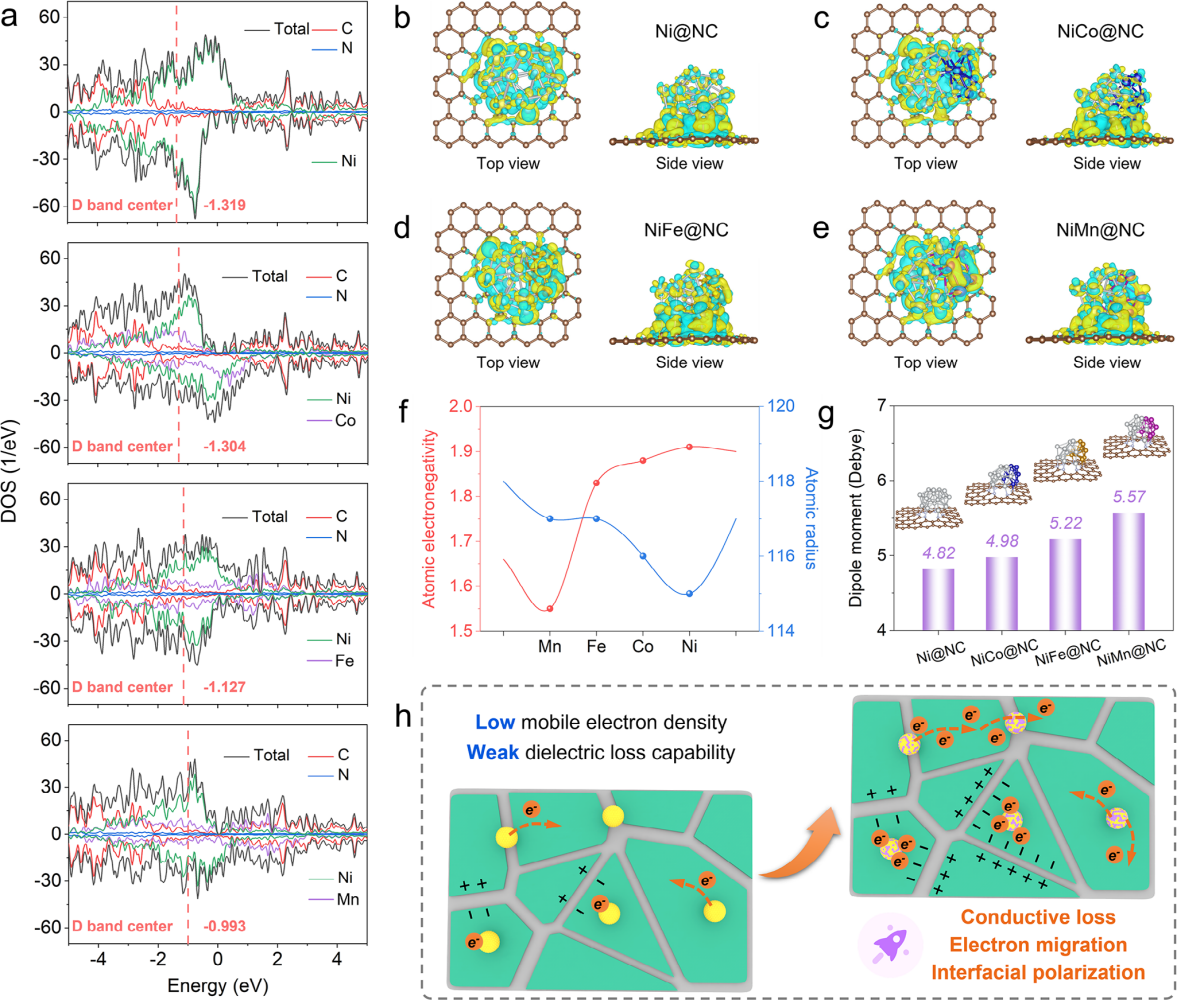
a) Partial density of states (PDOS) for Ni@NC and NiM@NC models. Charge density difference of b) Ni@NC, c) NiCo@NC, d) NiFe@NC and e) NiMn@NC. The yellow and indigo colors represent electron accumulation and depletion, respectively. f) Atomic electronegativities and radius for Ni, Co, Fe, and Mn atoms. g) Dipole moments of Ni@NC and NiM@NC systems. h) Dielectric loss mechanisms for Ni@NC and NiM@NC.

DFT simulations were subsequently performed to analyze the possible electron distribution and the induced dipole moments in NiM@NC systems, thus verifying the rationality and feasibility of the designed absorber system for electron cloud density modulation. In **Figure** **5**a, the D‐band center in NiCo@NC, NiFe@NC and NiMn@NC systems represent obvious positive shift compared to that of Ni@NC, suggesting stronger orbital hybridization effects between *d* electrons in alloy metal species and *p* electrons from C species near the Fermi energy level.^[^ ^42^ ^]^ This evolution leads to higher energy levels occupied by more electrons,^[^ ^43^, ^44^ ^]^ which effectively increases the electron density near the D band center and promotes electron hopping and rearrangement. The variations of electron cloud density in NiM@NC are further confirmed by the charge density difference results (Figure 5b–e). As observed in Ni@NC system in Figure 5b, significant electron enrichment (yellow color) emerges in the NC matrix adjacent to the Ni NPs, demonstrating that the spatial charges tend to flow from Ni atoms to N/C atoms. After the introduction of Co, Fe and Mn elements, the electron‐rich regions around the carbon layer are further enlarged. The electron accumulation and depletion regions within the NiM NPs appear on the Ni side and Co/Fe/Mn side, indicating that Ni atoms tend to gain electrons while Co/Fe/Mn atoms tend to donate electrons, which is consistent with the atomic electronegativity law in Figure 5f. The electron migration along the Ni‐N‐C path leads to localized electron cloud distortion, and the alloying treatment further amplifies the electron cloud asymmetry. As a result, larger dipole moments are formed in the NiM@NC system (Figure 5g). The increased dipole moments enable the formed dipoles to oscillate more violently in the presence of an external electromagnetic field, promoting the dissipation of EMW energy and inducing enhanced macroscopic polarization.

According to the above analysis, the underlying EMA mechanisms for Ni@NC and NiM@NC systems can be elucidated in the following aspects. In the Ni@NC system, the strong *2d*‐*3p* orbital hybridization between Ni and N/C atoms induces the generation of Ni‐N‐C electric dipoles, which contribute to the electron transfer from Ni to N/C atoms.^[^ ^45^ ^]^ As metal NPs present different intrinsic work functions from that of the carbon matrix, the abundant free electrons in Ni NPs will undergo accumulation and redistribution on contact surface of Ni/C. Consequently, the directional build‐in electric field will be developed, which in turn drives the charge hopping and migratory until dynamic equilibrium. Therefore, the Ni NPs loaded on the carbon layers can promote the localized electron migration and thus enhancing the polarization relaxation effect for Ni@NC systems. Moreover, Ni elements possess higher atomic electronegativity than that of Co/Fe/Mn elements. Therefore, Ni‐based alloy NPs obtained based on alloying treatment, especially NiFe and NiMn NPs, can serve as high‐density electron sources to provide abundant mobile electrons to the NC matrix. The rearrangement of mobile electrons at the edges of dielectric isolated islands leads to robust in‐plane interfacial polarization (Figure 5h), which improves the dielectric loss capability of the NiM@NC system. The enhanced local electron exchanges also improve the charge transmission efficiency of carbon substrates to a certain extent, and lead to the optimization of conductive losses induced by electron mobility as well. The optimized electronic structure promotes the dielectric polarization responses for NiM@NC systems at medium frequencies, especially in the C and X bands, enabling the EAB covering a wider range.

On the other hand, the incorporation of Co, Fe, and Mn elements can also tune the magnetic properties of Ni NPs and thus contribute to the impedance matching optimization.^[^ ^46^ ^]^ As decided in Table S1 (Supporting Information), the *M_s_* values for NiCo@NC and NiFe@NC increased from 31.3 emu g^−1^ of Ni@NC to 42.7 and 52.3 emu g^−1^, while that of NiMn@NC decreased to 19.6 emu g^−1^. As the atomic magnetic moments of Fe (≈2.2 µB) and Co (≈1.7 µB) are higher than that of Ni (≈0.6 µB) elements, the magnetic moments of Fe and Co in the Ni‐based alloy NPs can be coupled to those of Ni through exchange interactions, resulting in a stronger saturation magnetization moment. In contrast, the atomic magnetic moment of Mn is usually negative (≈−0.5 µB). Therefore, the anti‐parallel arrangement of the 3d orbital electron spins of Mn induces an antiferromagnetic coupling effect, leading to a lower *M_s_* value for NiMn alloy NPs. The modification in magnetic properties optimizes the magnetic responses of the alloy NPs under the external electromagnetic fields, resulting in better dielectric‐magnetic synergistic effects. The superior dielectric‐magnetic synergies enable NiFe@NC to exhibit optimal EMW energy conversion capabilities among Ni@NC and NiM@NC absorbers.

## 3 Conclusion

In summary, we prepared NC microdomain loaded with ≈40 nm Ni‐based alloy NPs to coordinate skin effect and dielectric loss performance. The NC layers were separated into numerous 50–100 nm dielectric isolated islands by the defect regions, thereby disrupting the continuous conductive network and avoiding the accumulation and reflection of the incident EMW in nanosheet surface. Meanwhile, the alloy NPs can serve as electronic sources to donate free electrons into the carbon support, contributing to the improvement of dielectric features. The electron cloud density of the Ni‐based alloys was controllably modulated by varying alloy species, including NiCo, NiFe and NiMn. The NiFe and NiMn NPs with stronger electron donating capabilities were able to offer more abundant mobile electrons into the isolated islands, inducing optimized dielectric loss performance by electron migration and in‐plane interfacial polarization. Accompanied with a better magnetic loss property, NiFe@NC with excellent magnetic‐dielectric synergy exhibited a RL_min_ value of −101.6 dB with a wide EAB up to 10.7 GHz. This work offers sufficient theoretical and experimental fundamentals for the design of high‐performance EMA systems with tunable electronic structures.

## Conflict of Interest

The authors declare no conflict of interest.

## Author Contributions

P.L. contributed to investigation (equal). D. Li contributed to conceptualization (equal) and writing – review & editing (lead). X.Z., Y.H., J.M., J.L., S.Q., and Z.S. contributed to investigation (supporting). Z.L. contributed to writing – review & editing (equal). Z.M. contributed to conceptualization (lead) and writing – review & editing (equal). S.M. contributed to conceptualization (lead) and writing – review & editing (equal).

## Supporting information



Supporting Information

## Data Availability

The data that support the findings of this study are available from the corresponding author upon reasonable request.

## References

[1] Z. Zhao , L. Zhang , H. Wu , Adv. Mater. 2022, 34, 2205376. 10.1002/adma.202205376 36067008

[2] Y. Han , H. Guo , H. Qiu , J. Hu , M. He , X. Shi , Y. Zhang , J. Kong , J. Gu , Adv. Funct. Mater. 2025, 2506803.

[3] M. Qin , L. Zhang , X. Zhao , H. Wu , Adv. Sci. 2021, 8, 2004640. 10.1002/advs.202004640 PMC8061380 33898201

[4] H. Shi , H. Gao , L. Qin , J. T. Yuan , L. X. Tang , Z. R. Wang , W. J. Li , Z. H. Feng , Y. Wang , A. M. Xie , Rare Met. 2025, 10.1007/s12598-025-03314-x.

[5] J. Liu , S. Zhang , D. Qu , X. Zhou , M. Yin , C. Wang , X. Zhang , S. Li , P. Zhang , Y. Zhou , K. Tao , M. Li , B. Wei , H. Wu , Nano‐Micro Lett. 2024, 17, 24. 10.1007/s40820-024-01515-0 PMC11436618 39331290

[6] S. Li , T. Xie , L. Ma , Z. Lei , N. Huang , H. Song , Y. Feng , B. Li , Y. Cui , L. Liu , W. Liu , B. Zhao , J. Zhang , R. Che , S. Ma , Z. Zhang , Carbon 2023, 213, 118302.

[7] M. Qin , L. Zhang , H. Wu , Adv. Sci. 2022, 9, 2105553. 10.1002/advs.202105553 PMC8981909 35128836

[8] C. Ding , C. Shao , Z. Wang , Z. Li , X. Guo , X. Ren , H. Pei , S. Wu , Q. Zhang , C. Wei , L. Xia , B. Zhong , G. Wen , X. Huang , Rare Met. 2025, 10.1007/s12598-025-03360-5.

[9] P. P. Zhou , C. Y. Hu , S. L. Yuan , J. C. Zhao , Y. W. Kuang , H. Gu , Y. S. Liu , L. X. Wang , Q. T. Zhang , Rare Met. 2025, 44, 4095.

[10] S. Wang , X. Zhang , S. Hao , J. Qiao , Z. Wang , L. Wu , J. Liu , F. Wang , Nano‐Micro Lett. 2023, 16, 16. 10.1007/s40820-023-01244-w PMC10656410 37975962

[11] F. Wang , Y. Liu , H. Zhao , L. Cui , L. Gai , X. Han , Y. Du , Chem. Eng. J. 2022, 450, 138160.

[12] Y. Shen , Z. Ma , F. Yan , C. Zhu , X. Zhang , Y. Chen , Adv. Funct. Mater. 2025, 35, 2423947.

[13] K. Zhang , Y. Yan , Z. Wang , G. Ma , D. Jia , X. Huang , Y. Zhou , Nano‐Micro Lett. 2024, 17, 46. 10.1007/s40820-024-01518-x PMC11489363 39422768

[14] Y. Hao , Z. Leng , C. Yu , P. Xie , S. Meng , L. Zhou , Y. Li , G. Liang , X. Li , C. Liu , Carbon 2023, 212, 118156.

[15] M. Qin , L. Zhang , X. Zhao , H. Wu , Adv. Funct. Mater. 2021, 31, 2103436.

[16] J. Tao , Y. Yan , J. Zhou , J. Wang , P. Chen , R. Tan , L. Xu , H. Zhu , W. Zhu , H. Huang , X. Tao , Z. Yao , Nat. Commun. 2025, 16, 3163. 40175363 10.1038/s41467-025-58448-4 PMC11965476

[17] J. Tao , K. Zou , J. Zhou , H. Wu , L. Xu , J. Wang , X. Tao , H. Huang , Z. Yao , Nat. Commun. 2024, 15, 10337. 39609414 10.1038/s41467-024-54770-5 PMC11604784

[18] W. Wang , R. Liu , J. Tao , T. Yu , Y. Liu , L. Duan , Z. Zhang , Z. He , S. Chen , J. Zhou , P. Chen , P. Liu , Z. Yao , Adv. Powder Mater. 2025, 4, 100302.

[19] J. Wang , C. Liang , X. Ma , P. Liu , W. Pan , H. Zhu , Z. Guo , Y. Sui , H. Liu , L. Liu , C. Yang , Adv. Mater. 2024, 36, 2307925. 10.1002/adma.202307925 37742133

[20] Y. Cheng , X. Liu , J. Ren , X. Xu , D. Lan , G. Wu , S. Zhang , Z. Gao , Z. Jia , G. Wu , Carbon 2025, 239, 120325.

[21] Z. Chen , G. Ding , Z. Wang , Y. Xiao , X. Liu , L. Chen , C. Li , H. Huang , G. Liao , Adv. Funct. Mater. 2025, 35, 2423213.

[22] Q. Xiao , W. Li , S. Xie , L. Wang , C. Y. Tang , Nat. Commun. 2024, 15, 9607. 39505857 10.1038/s41467-024-54055-x PMC11541988

[23] G. X. Liu , K. Zhang , C. Han , X. L. Shao , J. Lu , L. Zhang , X. S. Jiang , Z. X. Wu , L. Yang , Tungsten 2024, 6, 767.

[24] P. Scotland , L. Eddy , J. Chen , W. Chen , J. L. Beckham , K. M. Wyss , C. H. Choi , P. A. Advincula , A. Lathem , O. E. Onah , Y. Han , J. M. Tour , ACS Nano 2025, 19, 11987. 40117566 10.1021/acsnano.4c16959

[25] Z. Shi , Y. Zhang , W. Guo , Z. Niu , Y. Chen , J. Huang , Adv. Funct. Mater. 2025, 35, 2414935.

[26] Y. Shi , Z. Ma , X. Zhang , F. Yan , Y. Zhao , C. Zhu , Y. Chen , Adv. Funct. Mater. 2024, 34, 2313483.

[27] Z. Ma , Y. Shen , X. Zhang , B. Li , Y. Chen , C. Zhu , Adv. Funct. Mater. 2025, 35, 2413784.

[28] X. Zhang , B. Li , J. Xu , X. Zhang , Y. Shi , C. Zhu , X. Zhang , Y. Chen , Adv. Funct. Mater. 2023, 33, 2210456.

[29] M. Li , H. Zhu , Q. Yuan , T. Li , M. Wang , P. Zhang , Y. Zhao , D. Qin , W. Guo , B. Liu , X. Yang , Y. Liu , Y. Pan , Adv. Funct. Mater. 2023, 33, 2210867.

[30] L. Huang , F. Tang , P. Liu , W. Xiong , S. Jia , F. Hao , Y. Lv , H. Luo , Fuel 2022, 327, 125115.

[31] W. Guo , C. Dun , C. Yu , X. Song , F. Yang , W. Kuang , Y. Xie , S. Li , Z. Wang , J. Yu , G. Fu , J. Guo , M. A. Marcus , J. J. Urban , Q. Zhang , J. Qiu , Nat. Commun. 2022, 13, 1409. 35301288 10.1038/s41467-022-28918-0 PMC8931012

[32] J. Wei , P. Gao , S. Wang , Y. Ma , D. Cao , D. Cheng , ACS Catal. 2025, 15, 1399.

[33] K. Huang , R. Li , H. Qi , S. Yang , S. An , C. Lian , Q. Xu , H. Liu , J. Hu , ACS Catal. 2024, 14, 8889.

[34] J. Hu , X. Xu , Y. Ji , H. Zhu , Y. Zhou , M. Zhu , Y. Huang , Appl. Catal. B‐Environ. 2025, 373, 125337.

[35] X. Liu , J. Zhou , Y. Xue , X. Lu , Nano‐Micro Lett. 2024, 16, 174. 10.1007/s40820-024-01396-3 PMC11018581 38619635

[36] X. Wang , Q. Li , H. Lai , Y. Peng , C. Hou , S. Xu , Adv. Funct. Mater. 2025, 2425949.

[37] Y. Li , X. Chen , Q. Wei , W. Liu , Y. Zhang , G. Qin , Z. Shi , X. Zhang , Sci. Bull. 2020, 65, 623. 10.1016/j.scib.2020.01.009 36659131

[38] X. Su , J. Wang , T. Liu , Y. Zhang , Y. Liu , B. Zhang , Y. Liu , H. Wu , H. X. Xu , Adv. Funct. Mater. 2024, 34, 2403397.

[39] P. Wu , X. Kong , Y. Feng , W. Ding , Z. Sheng , Q. Liu , G. Ji , Adv. Funct. Mater. 2024, 34, 2311983.

[40] J. Du , T. Li , J. Li , J. Tang , R. Zhang , Y. Liu , J. Feng , F. Meng , Adv. Fiber Mater. 2025, 7, 811.

[41] D. Li , H. Liao , H. Kikuchi , T. Liu , ACS Appl. Mater. Interfaces 2017, 9, 44704. 29199817 10.1021/acsami.7b13538

[42] H. Long , X. Zhang , Z. Zhang , J. Zhang , J. Yu , H. Yu , Nat. Commun. 2025, 16, 946. 39843935 10.1038/s41467-025-56306-x PMC11754433

[43] X. Song , J. Wang , Q. Jiang , M. Li , R. Duan , J. Li , W. Li , W. Xiao , G. Zhang , C. Xie , X. Sun , X. Li , Adv. Funct. Mater. 2025, 2502181.

[44] R. Zeng , Q. Gao , L. Xiao , W. Wang , Y. Gu , H. Huang , Y. Tan , D. Tang , S. Guo , J. Am. Chem. Soc. 2024, 146, 10023. 38554097 10.1021/jacs.4c00791

[45] S. Cheng , D. Sheng , S. Mukherjee , W. Dong , Y. Huang , R. Cao , A. Xie , R. A. Fischer , W. Li , Nat. Commun. 2024, 15, 9077. 39433804 10.1038/s41467-024-53465-1 PMC11494010

[46] L. Liang , Q. Li , X. Yan , Y. Feng , Y. Wang , H. B. Zhang , X. Zhou , C. Liu , C. Shen , X. Xie , ACS Nano 2021, 15, 6622. 33780231 10.1021/acsnano.0c09982

