# Supplementary material for: Tunable Ni‐Based Alloys as Electrons Donor in Dielectric Isolated Islands to Achieve Broad Frequency Electromagnetic Wave Absorption
Source: Adv Sci (Weinh). 2025 Jul 16;12(38):e10198. doi: 10.1002/advs.202510198 (PMC12520480; doi:10.1002/advs.202510198)
Supplement: Supplementary file 1 — Supporting Information [file ADVS-12-e10198-s001.docx]

**Supporting information**

**Tunable Ni-based alloys as electrons donor in dielectric isolated islands to achieve broad frequency electromagnetic wave absorption**

Pengfei Liu^a^, Da Li^a^*, Xiaoguang Zhao^b^, Yanqi Huang^a^, Jingyun Ma^a^, Jiangfang Lian^a^, Shijie Qiu^a^, Zhisen Shen^a^, Zhuan Li^c^*, Zhenhui Ma^d^*, Song Ma^e^*

*^a^The Affiliated Lihuili Hospital of Ningbo University, Ningbo, 315040, China*

*^b^Department of Electrical and Electronic Engineering, The University of Hong Kong, Hong Kong, SAR, China*

*^c^State Key Laboratory of Powder Metallurgy, Central South University, Changsha 410083, PR China*

*^d^Department of Physics, Beijing Technology and Business University, Beijing, 100048, China*

*^e^Shenyang National Laboratory for Materials Science, Institute of Metal Research, Chinese Academy of Sciences, 110016, China*

*Corresponding author

*Email address*:

[lhllida@nbu.edu.cn](mailto:lhllida@nbu.edu.cn); [lizhuan@csu.edu.cn](mailto:lizhuan@csu.edu.cn); [mazh@btbu.edu.cn](mailto:mazh@btbu.edu.cn); [songma@imr.ac.cn](mailto:songma@imr.ac.cn)

**Experimental section**

*Materials*: Dopamine hydrochloride (DA, 98%), Cobalt nitrate hexahydrate (Co(NO_3_)_2_·6H_2_O, 99%), Iron(III) nitrate nonahydrate (Fe(NO_3_)_3_·9H_2_O, 98.5%), Manganese(II) nitrate tetrahydrate (Mn(NO_3_)_2_·4(H_2_O), 98%) were purchased from Maclin. Nickel nitrate hexahydrate (NiN_2_O_6_·6H_2_O, AR) was purchased from Aladdin. And Hydrogen peroxide solution (H_2_O_2_, 30 wt%) were purchased from Shanghai Maokang Biotechnology Co., Ltd. All the reagents were used directly without further purification.

*Preparation of Ni@NC*: Briefly, 0.8 g of DA and 0.6 g of NiN_2_O_6_·6H_2_O were mixed together and grinded thoroughly for 10 min. 1 mL of H_2_O_2_ was further added to the mixture drop by drop, accompanied by grinding for 5 min. The mixture was then transferred to an oven overnight at 60 ℃ to allow complete drying. Subsequently, the dry mixture was put into a vacuum tube furnace for carbonization under Ar atmosphere with a flow of 200 sccm. After a two-step heating process (first warmed up to 200 ℃ with 2 ℃/min, then heated up to 800 ℃ with 5 ℃/min) and held at 800 ℃ for 2h, the obtained black powder was denoted as Ni@NC.

*Preparation of NiM@NC*: The NiM@NC composites were prepared following similar approaches to that of Ni@NC, except replacing the NiN_2_O_6_·6H_2_O with the mixture of NiN_2_O_6_·6H_2_O and Co(NO_3_)_2_·6H_2_O/Fe(NO_3_)_3_·9H_2_O/Mn(NO_3_)_2_·4(H_2_O). The molar ratio of the added Ni and Co/Fe/Mn was set at 4 : 1. The obtained products were named as NiCo@NC, NiFe@NC and NiMn@NC, respectively.

*Characterizations*: The composition and graphitization of the prepared Ni@NC and NiM@NC were recorded with X-ray diffraction spectra (XRD, Rigaku SmartLab SE) with Cu Kα radiation source and Raman spectroscopy (Thermo Scientific DXR), respectively. The surface chemical information was revealed by X-ray photoelectron spectroscopic (XPS, Krayos AXIS Ultra DLD) measurements with an Al Kα X-ray source. Field-emission transmission electron microscopy (TEM, JEM-F200) were conducted for the microstructure and morphology analysis. The saturation magnetization (*M_s_*) and coercivity (*H_c_*) of the obtained composites were recorded by vibrating sample magnetometer (VSM, LakeShore 7404). The coordination information of Ni single atoms were explored by X-ray absorption spectroscopy (XAS) at the Shanghai Synchrotron Radiation Facility (SSRF) with 02B02 beamline.

*Electromagnetic Measurement*: The EM parameters for Ni@NC and NiM@NC composites with a loading content of 20 wt% were recorded by vector network analyzer (VNA, Agilent E5071c). Toroidal ring samples (7.00 mm for outer diameter, 3.04 mm for inner diameter, 2.00 mm for the thickness) were used for the EM measurements. The reflection loss values of the absorbers were evaluated based on the transmission line theory, which can be presented by the following equations:^[^[^1^](#_ENREF_1)^]^

$Z_{in}=Z_{0}\sqrt{\frac{\mu_{r}}{\varepsilon_{r}}}\tan h\left[ j\left( \frac{2\pi fd}{c} \right)\sqrt{\varepsilon_{r}\mu_{r}} \right]$ (1)

$RL=20\log_{10} \frac{\left| Z_{in}-Z_{0} \right|}{\left| Z_{in}+Z_{0} \right|}$ (2)

where *Z*_in_ and *Z*_0_ represent the input impedance and the free space impedance, respectively. *ε_r_* is the complex permittivity and *μ*_r_ is the complex permeability. *f*, *d* and *c* represent the EMW frequency, the absorber thickness, and the speed of light, respectively.

The attenuation constants (*α*) for the obtained absorber systems were calculated based on the following equation:^[^[^1^](#_ENREF_1)^]^

$\alpha=\text{ }\frac{\sqrt{2}\pi f}{c}\sqrt{\left(\mu''\varepsilon''-\mu'\varepsilon'\right)+\sqrt{(\mu''\varepsilon''-\mu'\varepsilon')^{2}+(\mu'\varepsilon''+\mu''\varepsilon')^{2}}}$ (3)

where *f* and *c* present the EMW frequency and vacuum velocity of light, respectively.

*RCS simulation*: The radar cross section (RCS) simulation were conducted using CST Studio Suite 2021. The calculation model was set as 180 × 180 mm^2^ with a perfect electric conductor (PEC) layer as the substrate (1.0 mm). The top layers were coated by the as-prepared Ni@NC and NiM@NC composites. The RCS signals under the far-field excitation can be evaluated based on the following equation:^[^[^2^](#_ENREF_2)^]^

$\sigma\left( dB m^{2} \right)=10log((4\pi S/\lambda^{2})\mid E_{s}/E_{i}\mid)$ (4)

Where *S* is the area of the simulation model, *λ* is the wavelength of EM wave, *Es* and *Ei* represent the electric field intensity of the scattered wave and incident wave, respectively.

*DFT simulation*: The spin-polarized density functional theory (DFT) calculations within the generalized gradient approximation (GGA) were performed based on the Perdew-Burke- Ernzerhof (PBE) formulation.^[^[^3^](#_ENREF_3)^]^ The projected augmented wave (PAW) potentials^[^[^4^](#_ENREF_4)^,^[^5^](#_ENREF_5)^]^ were chosen to describe the ionic cores and valence electrons were considered using a plane wave basis set with a kinetic energy cutoff of 400eV. Partial occupancies of the Kohn−Sham orbitals were allowed using the Gaussian smearing method and a width of 0.05 eV. The electronic energy was considered self-consistent when the energy change was smaller than 10^−6^ eV. A geometry optimization was considered convergent when the force change was smaller than -0.05 eV/Å^2^. Grimme’s DFT-D3 methodology was used to describe the dispersion interactions among all the atoms.^[^[^6^](#_ENREF_6)^]^ During structural optimizations of the surface models, the 2×2×1 gamma-point centered k-point grid for Brillouin zone was used.


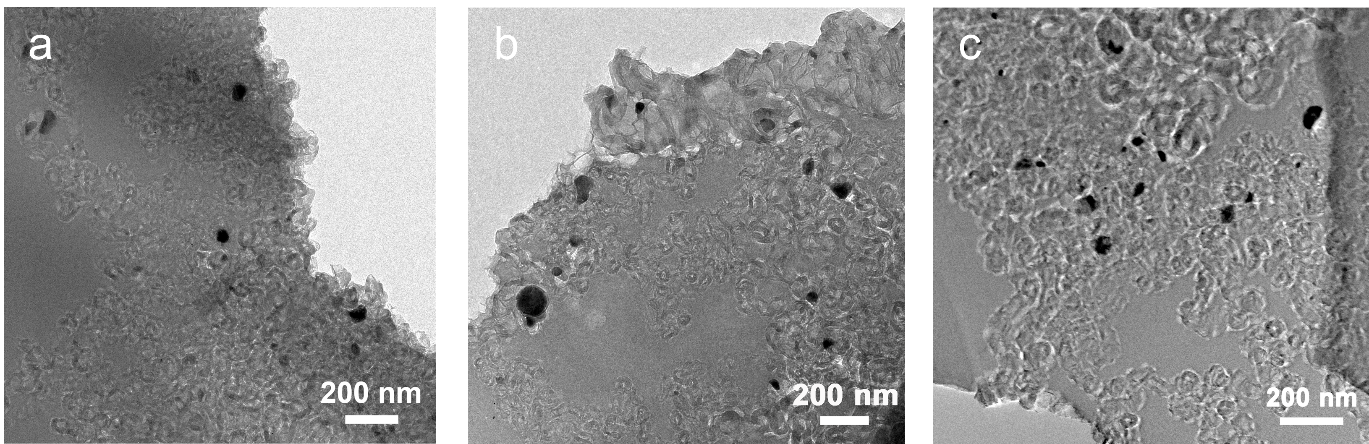


**Figure S1.** TEM images of a) Ni@NC, b) NiCo@NC and c) NiMn@NC composites.


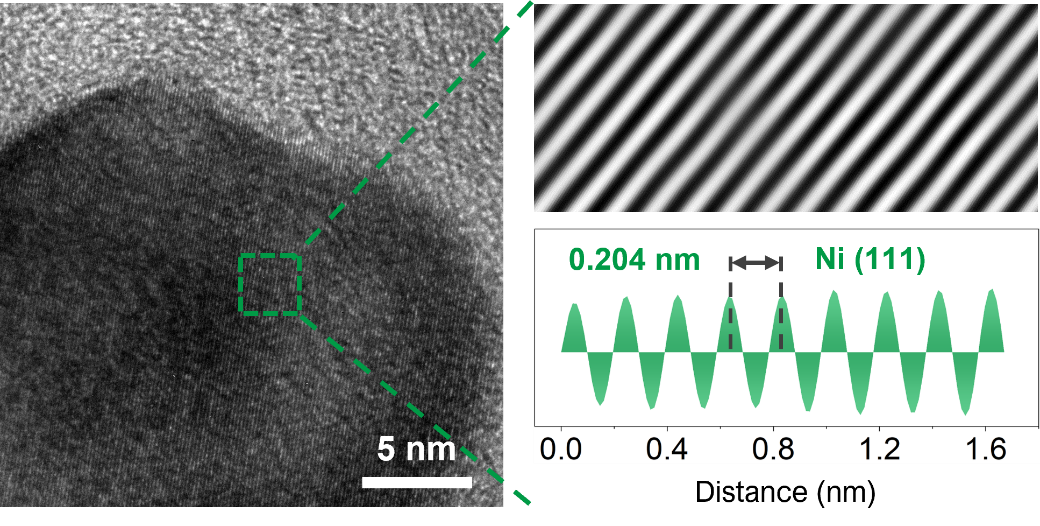


**Figure S2.** HRTEM image and the corresponding FFT converted lattice fringes of Ni NPs in Ni@NC composite.


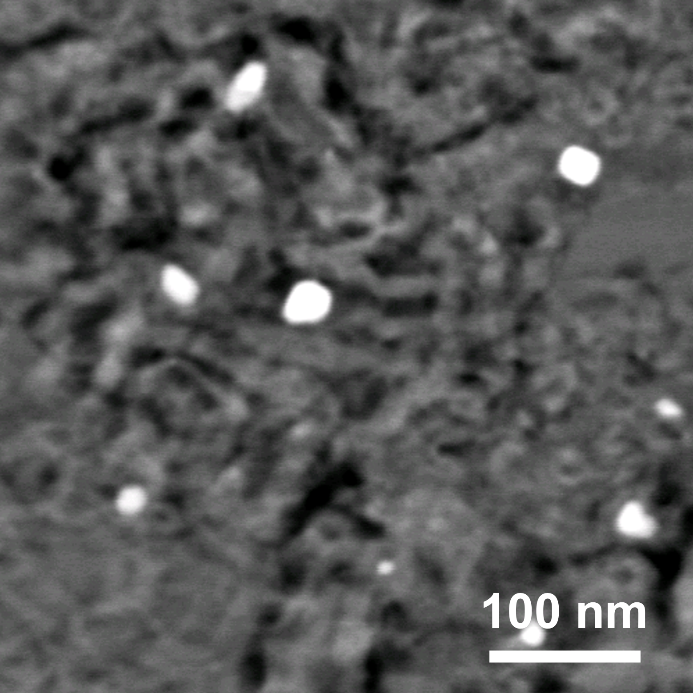


**Figure S3.** Dark field STEM image of NiFe@NC corresponded to the rough morphology.


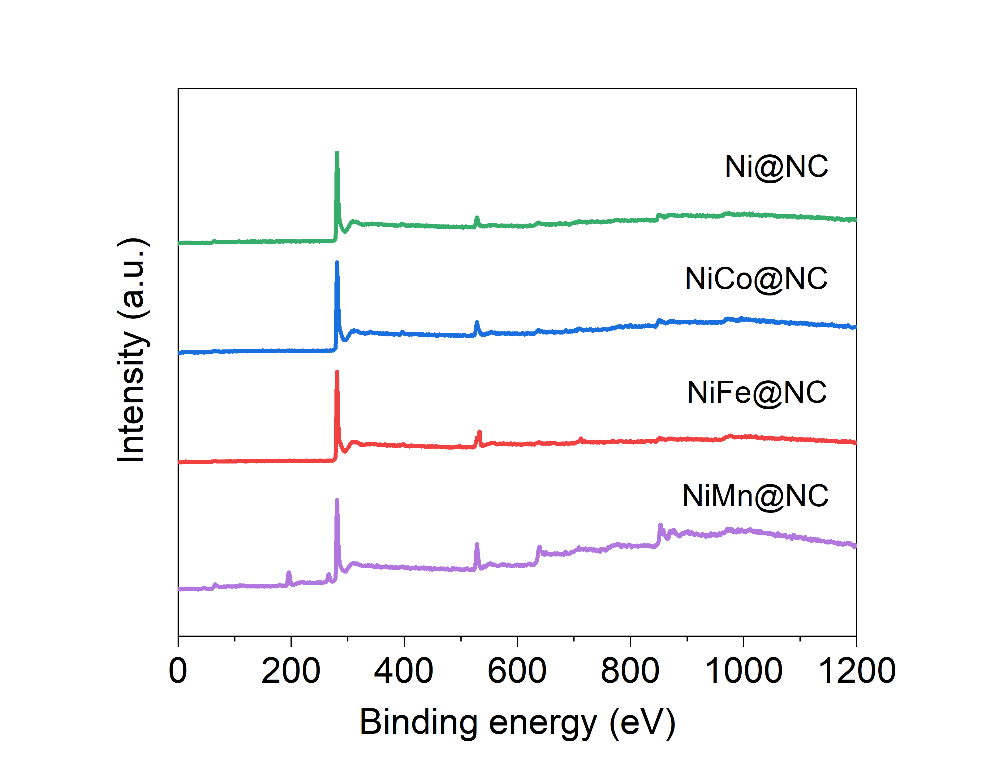


**Figure S4.** Wide XPS spectra of Ni@NC and NiM@NC composites.


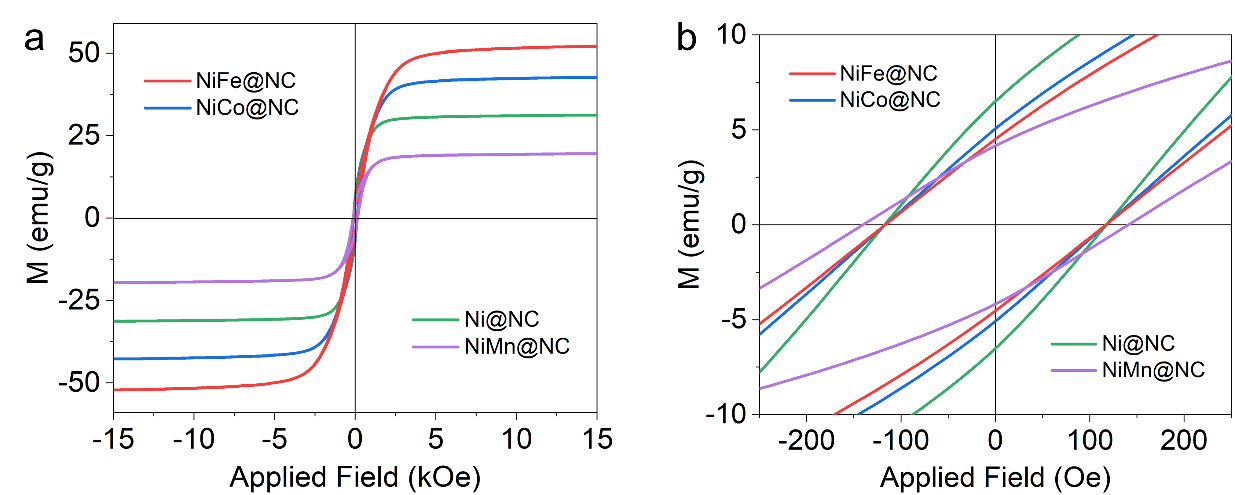


**Figure S5.** Magnetic hysteresis loops of Ni@NC and NiM@NC composites.


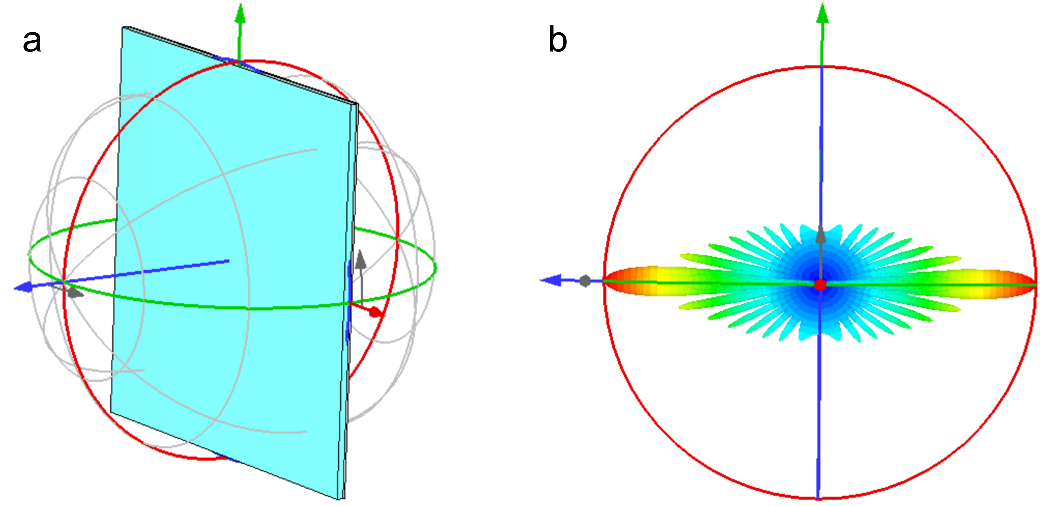


**Figure S6.** a) Simulation models for CST calculation and b) 3D RCS signal PEC.


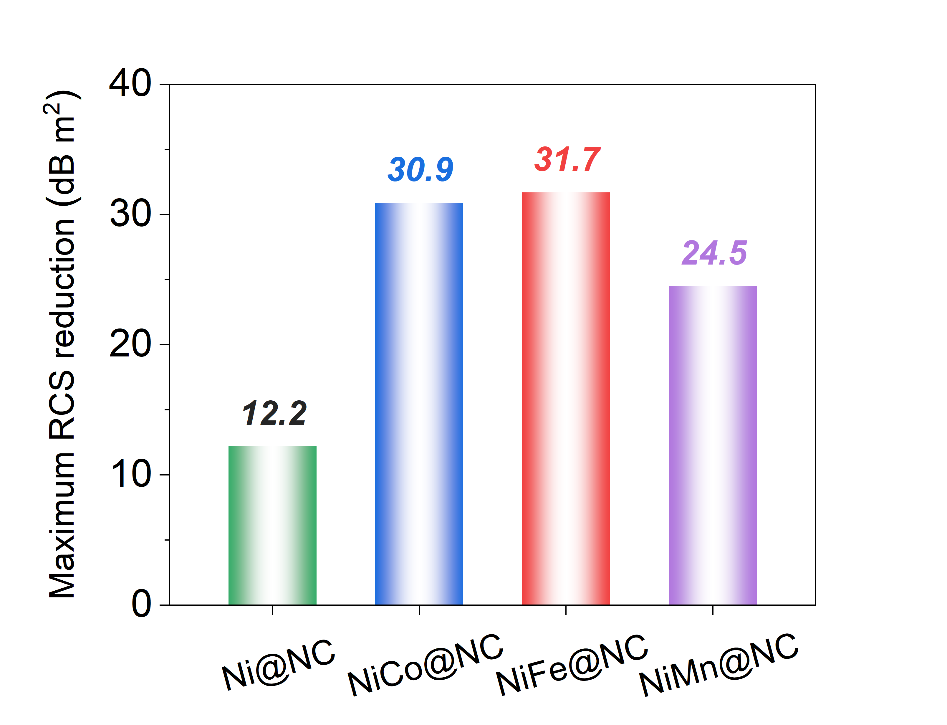


**Figure S7.** Maximum RCS signal reduction of Ni@NC and NiM@NC composites.


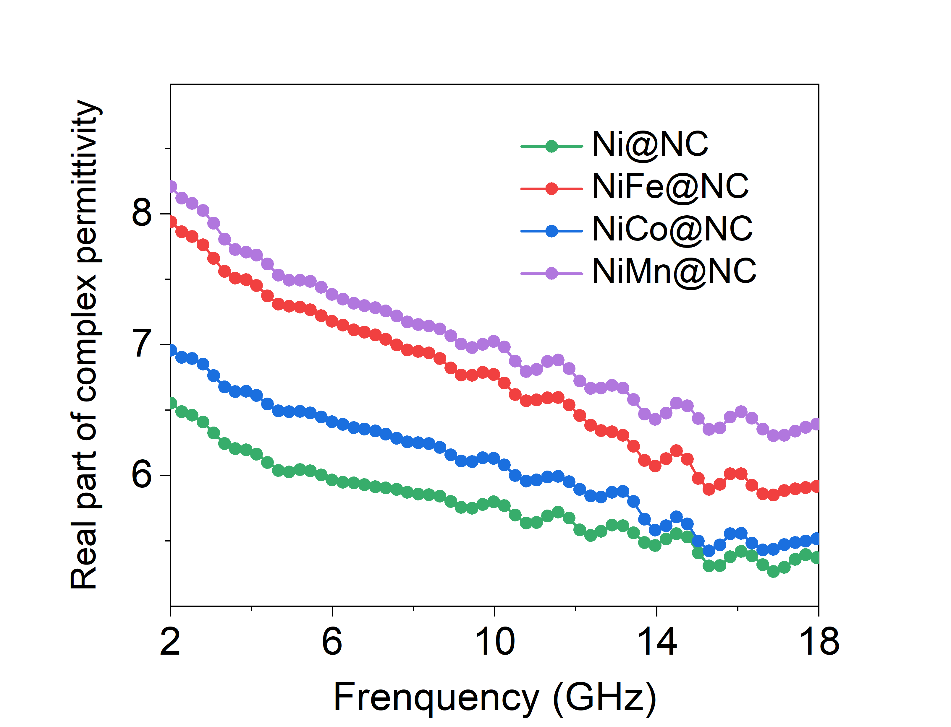


**Figure S8.** Real part of the complex permittivity for Ni@NC and NiM@NC.


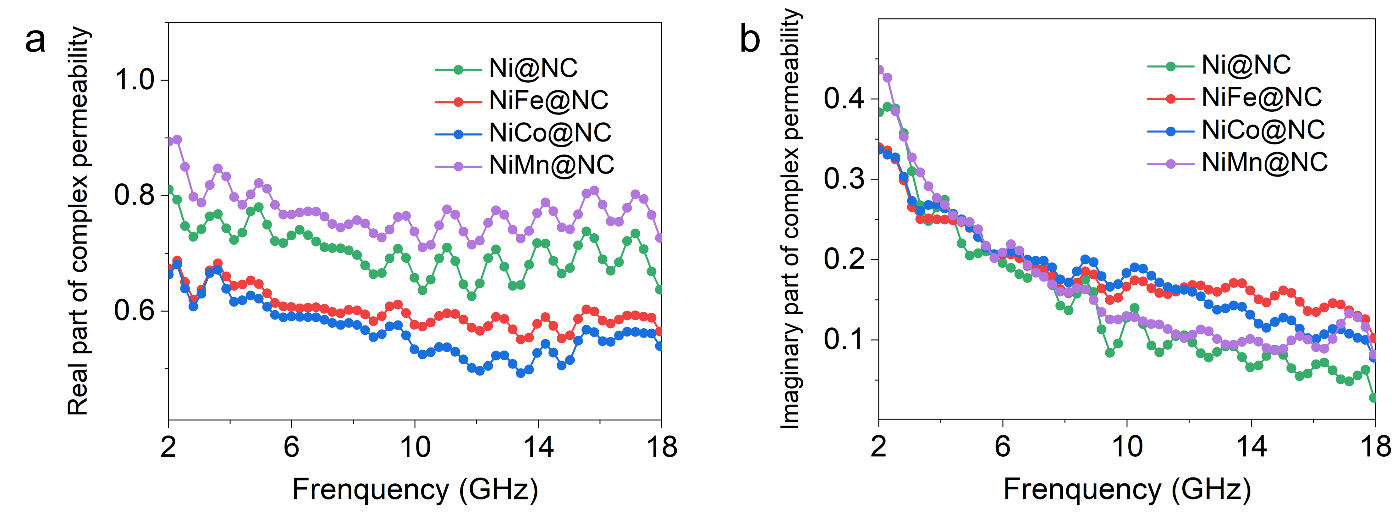


**Figure S9.** a) Real and b) imaginary part of the complex permeability for Ni@NC and NiM@NC.


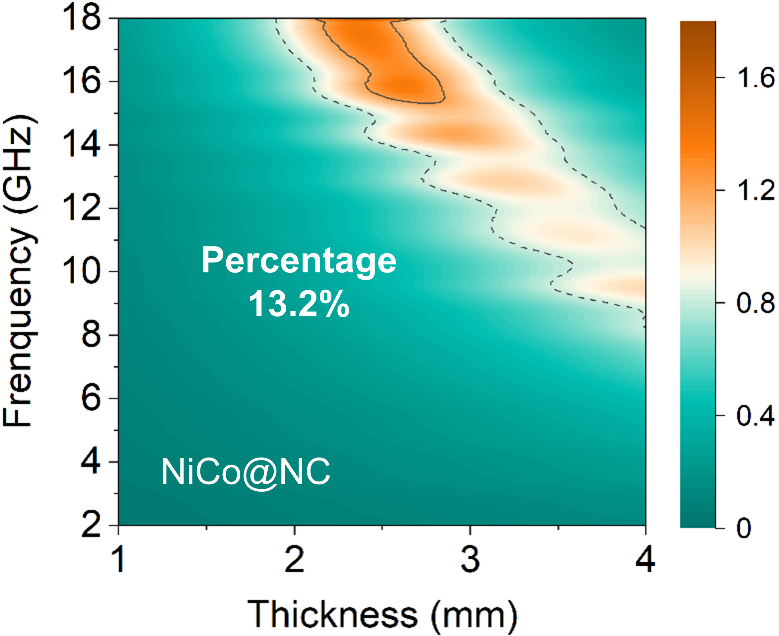


**Figure S10.** 2D impedance matching plots for NiCo@NC.

**Table S1.** Summary of the material properties for Ni@NC and NiM@NC samples.

| **Absorber**  **systems** | **Particle size**  **(nm)** | ***M_s_***  **(emu/g)** | ***H_c_***  **(Oe)** | **Dipole**  **moment**  **(Debye)** | **RL_min_ (dB)** | **EAB (GHz)** | **Matching**  **thickness (mm)** |
| --- | --- | --- | --- | --- | --- | --- | --- |
| **Ni@NC** | 34.2 | 31.3 | 121.1 | 4.82 | -42.5 | 7.2 | 2.8 |
| **NiCo@NC** | 38.6 | 42.7 | 121.2 | 4.98 | -110.4 | 9.2 | 3.1 |
| **NiFe@NC** | 36.2 | 52.3 | 121.1 | 5.22 | -101.6 | 10.7 | 3.2 |
| **NiMn@NC** | 37.3 | 19.6 | 141.2 | 5.57 | -71.5 | 9.6 | 2.7 |

**Table S2.** Summary on the EMA performance of the reported absorber systems and FeNC-NO_3_ in this work.

| **EMA systems** | **Absorbers** | **RL_min_ (dB)** | **EAB (GHz)** | **Ref.** |
| --- | --- | --- | --- | --- |
| **Reported**  **absorber**  **systems** | CoNi@NC | -59.9 | 5.6 | [[7](#_ENREF_7)] |
|  | Ni/MXene | -62.7 | 6.9 | [[1](#_ENREF_1)] |
|  | Ni-HMCNTs | -63.5 | 5.1 | [[2](#_ENREF_2)] |
|  | Ni_3_Fe@CNT | -57.3 | 6.0 | [[8](#_ENREF_8)] |
|  | Ni/CNFs | -60.1 | 7.6 | [[9](#_ENREF_9)] |
|  | Ni_2_P/SCF | -56.9 | 7.2 | [[10](#_ENREF_10)] |
|  | RENi-MOF | -61.6 | 5.1 | [[11](#_ENREF_11)] |
|  | Ni/MnO@C | -64.1 | 7.4 | [[12](#_ENREF_12)] |
|  | Fe_2_N@Ni3Fe/CNF | -56.8 | 6.7 | [[13](#_ENREF_13)] |
|  | Ce/NiCo@C | -67.2 | 7.1 | [[14](#_ENREF_14)] |
|  | Fe_4_N@NGC/Ce | -85.1 | 7.5 | [[15](#_ENREF_15)] |
|  | CSC aerogel | -52.8 | 8.8 | [[16](#_ENREF_16)] |
|  | 3D N/S-HPC | -60.3 | 7.4 | [[17](#_ENREF_17)] |
|  | Co-N-C/ZrO_2_ | -69.7 | 4.9 | [[18](#_ENREF_18)] |
|  | Co-SAs@NC | -54.4 | 8.4 | [[19](#_ENREF_19)] |
|  | CEG-6 | -72.3 | 4.1 | [[20](#_ENREF_20)] |
|  | B-NCP | -52.0 | 5.4 | [[21](#_ENREF_21)] |
|  | ZIF–MXene | -47.4 | 6.3 | [[22](#_ENREF_22)] |
|  | M-CAs | -48.3 | 6.8 | [[23](#_ENREF_23)] |
| **NiM@NC**  **systems** | NiCo@NC | -110.4 | 9.2 | This work |
|  | NiFe@NC | -101.6 | 10.7 |  |
|  | NiMn@NC | -71.5 | 9.6 |  |

**References**

[1] H. Cheng, Y. Pan, X. Wang, C. Liu, C. Shen, D. W. Schubert, Z. Guo, X. Liu, *Nano-Micro Lett.* **2022**, *14*, 63.

[2] L. Chen, J. Pan, T. Wang, W. Xia, J. He, K. Zhang, *Adv. Funct. Mater.* **2025**, *35*, 2409432.

[3] J. P. Perdew, K. Burke, M. Ernzerhof, *Phys. Rev. Lett.* **1996**, *77*, 3865-3868.

[4] G. Kresse, D. Joubert, *Phys. Rev. B* **1999**, *59*, 1758-1775.

[5] P. E. Blöchl, *Phys. Rev. B* **1994**, *50*, 17953-17979.

[6] S. Grimme, J. Antony, S. Ehrlich, H. Krieg, *J. Chem. Phys.* **2010**, *132*,

[7] Q. Liang, M. He, B. Zhan, H. Guo, X. Qi, Y. Qu, Y. Zhang, W. Zhong, J. Gu, *Nano-Micro Lett.* **2025**, *17*, 167.

[8] S. Li, T. Xie, L. Ma, Z. Lei, N. Huang, H. Song, Y. Feng, B. Li, Y. Cui, L. Liu, W. Liu, B. Zhao, J. Zhang, R. Che, S. Ma, Z. Zhang, *Carbon* **2023**, *213*, 118302.

[9] X. Meng, M. Yu, C. Wang, *Adv. Fiber Mater.* **2025**, *7*, 469-480.

[10] F. Pan, L. Cai, Y. Dong, X. Zhu, Y. Shi, W. Lu, *J. Mater. Sci .Technol.* **2022**, *101*, 85-94.

[11] W. Zhang, G. Tan, J. Hu, Q. Wang, W. Yan, Q. Man, *Chem. Eng. J.* **2023**, *478*, 147414.

[12] S. Wang, X. Zhang, S. Hao, J. Qiao, Z. Wang, L. Wu, J. Liu, F. Wang, *Nano-Micro Lett.* **2023**, *16*, 16.

[13] S. Xu, Z. Jia, D. Lan, Z. Gao, S. Zhang, G. Wu, *Adv. Funct. Mater.* **2025**, *n/a*, 2500304.

[14] W. Yan, J. Hu, C. Yi, Q. Wang, J. Wang, W. Zhang, X. Huang, Y. Liu, X. Gao, H. Zeng, F. Yang, G. Tan, X. Chi, *Adv. Funct. Mater.* **2025**, *n/a*, 2501645.

[15] Z. Ma, K. Yang, D. Li, H. Liu, S. Hui, Y. Jiang, S. Li, Y. Li, W. Yang, H. Wu, Y. Hou, *Adv. Mater.* **2024**, *36*, 2314233.

[16] X. Su, J. Wang, T. Liu, Y. Zhang, Y. Liu, B. Zhang, Y. Liu, H. Wu, H.-X. Xu, *Adv. Funct. Mater.* **2024**, *34*, 2403397.

[17] Z. Wei, X. Chen, D. Chen, J. Liang, Z. Liao, X. Li, Z. Li, J. Kong, *Carbon* **2025**, *233*, 119925.

[18] L. Zhou, P. Hu, M. Bai, N. Leng, B. Cai, H.-L. Peng, P.-Y. Zhao, Y. Guo, M. He, G.-S. Wang, J. Gu, *Adv. Mater.* **2025**, *37*, 2418321.

[19] B. Wang, W. Wei, F. Huang, F. Liu, S. Li, H. Zhang, *Adv. Funct. Mater.* **2024**, *34*, 2404484.

[20] Z. Tang, L. Xu, C. Xie, L. Guo, L. Zhang, S. Guo, J. Peng, *Nat. Commun.* **2023**, *14*, 5951.

[21] A. Cui, C. Wang, Y. Miao, X. Wang, Y. Wang, D. Lan, S. Wu, G. Song, T. Wang, Z. Tian, H. Wu, Z. Li, M. Zhang, *Adv. Funct. Mater.* **2025**, *35*, 2420292.

[22] Z. Gao, A. Iqbal, T. Hassan, S. Hui, H. Wu, C. M. Koo, *Adv. Mater.* **2024**, *36*, 2311411.

[23] J. Liu, S. Zhang, D. Qu, X. Zhou, M. Yin, C. Wang, X. Zhang, S. Li, P. Zhang, Y. Zhou, K. Tao, M. Li, B. Wei, H. Wu, *Nano-Micro Lett.* **2024**, *17*, 24.
